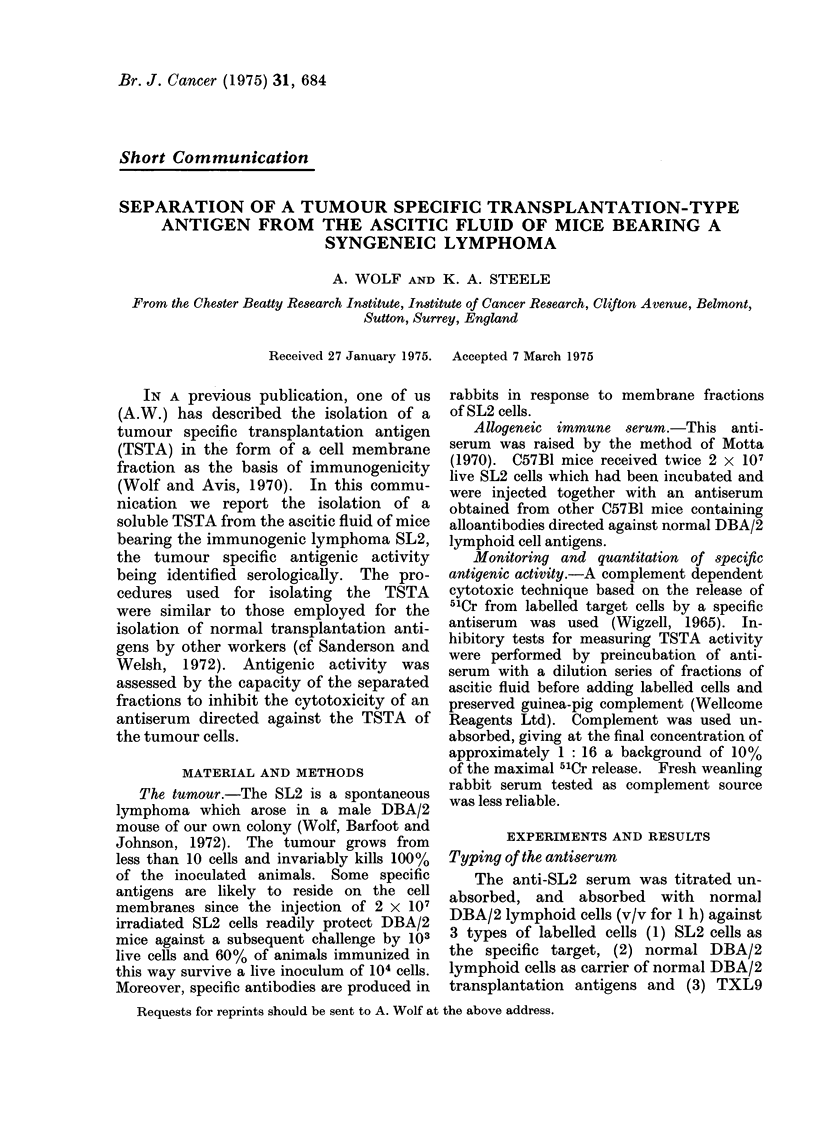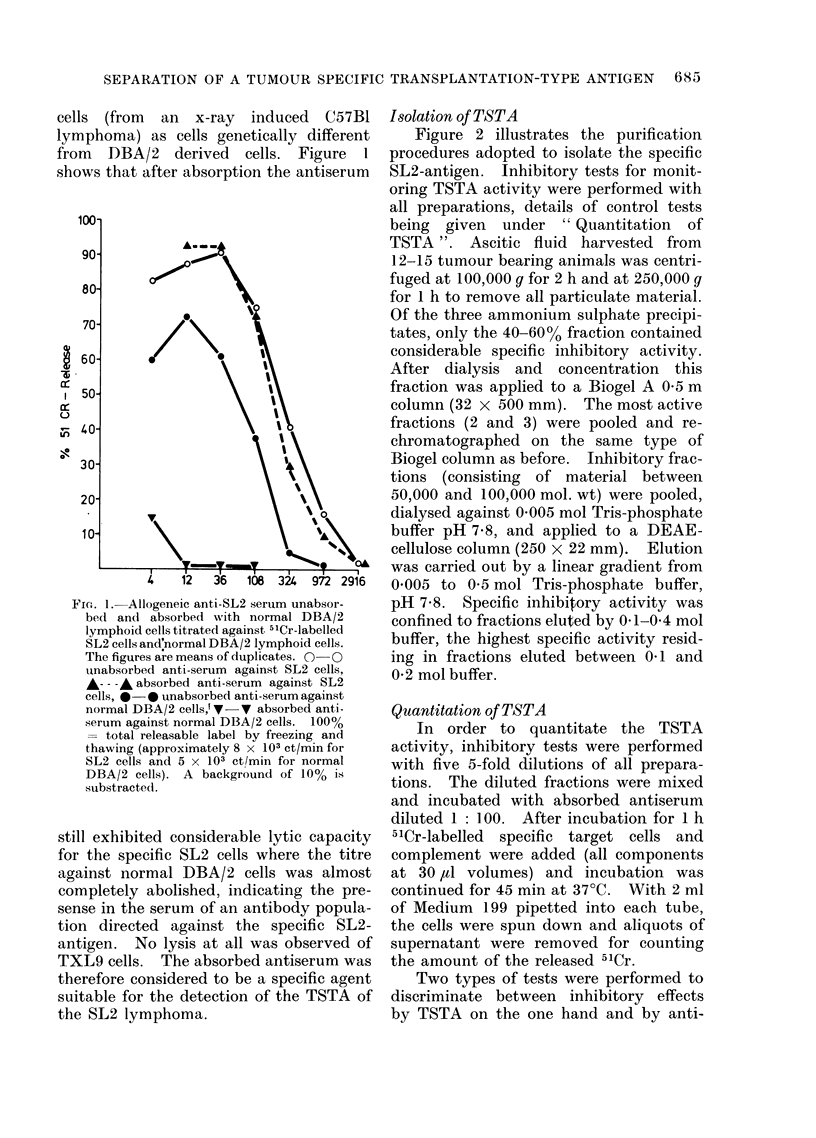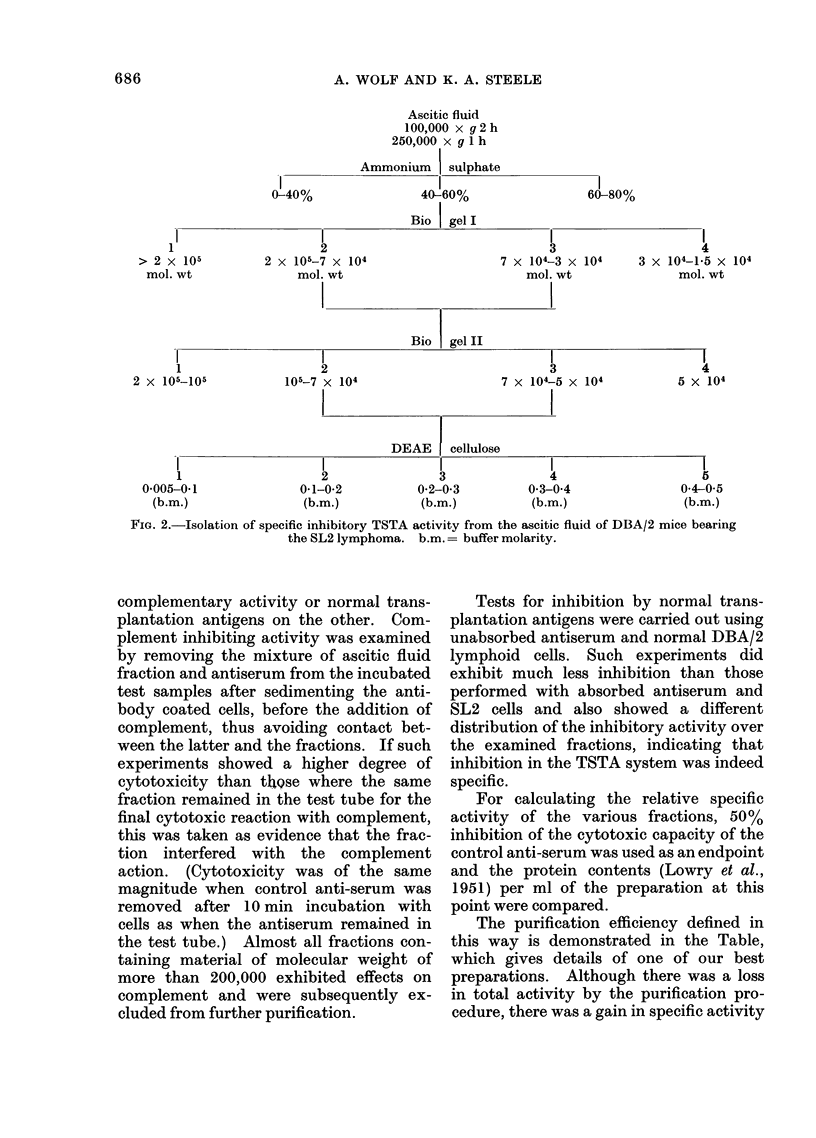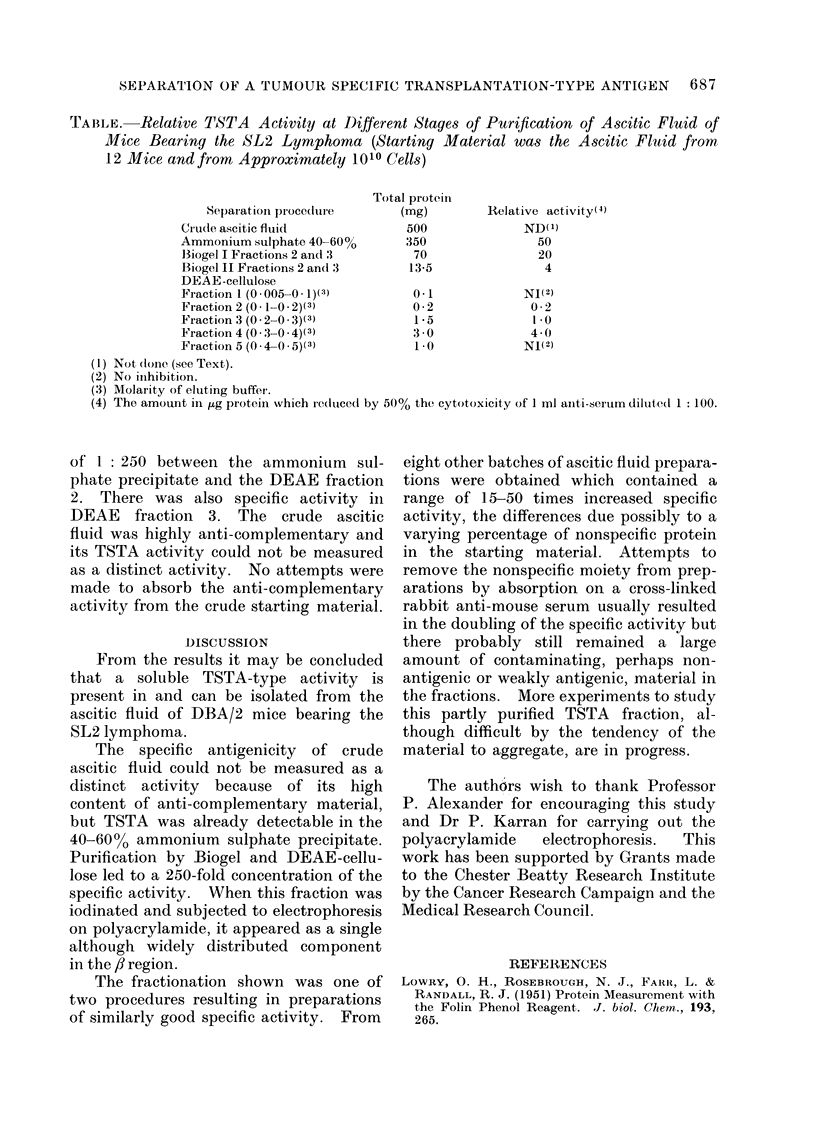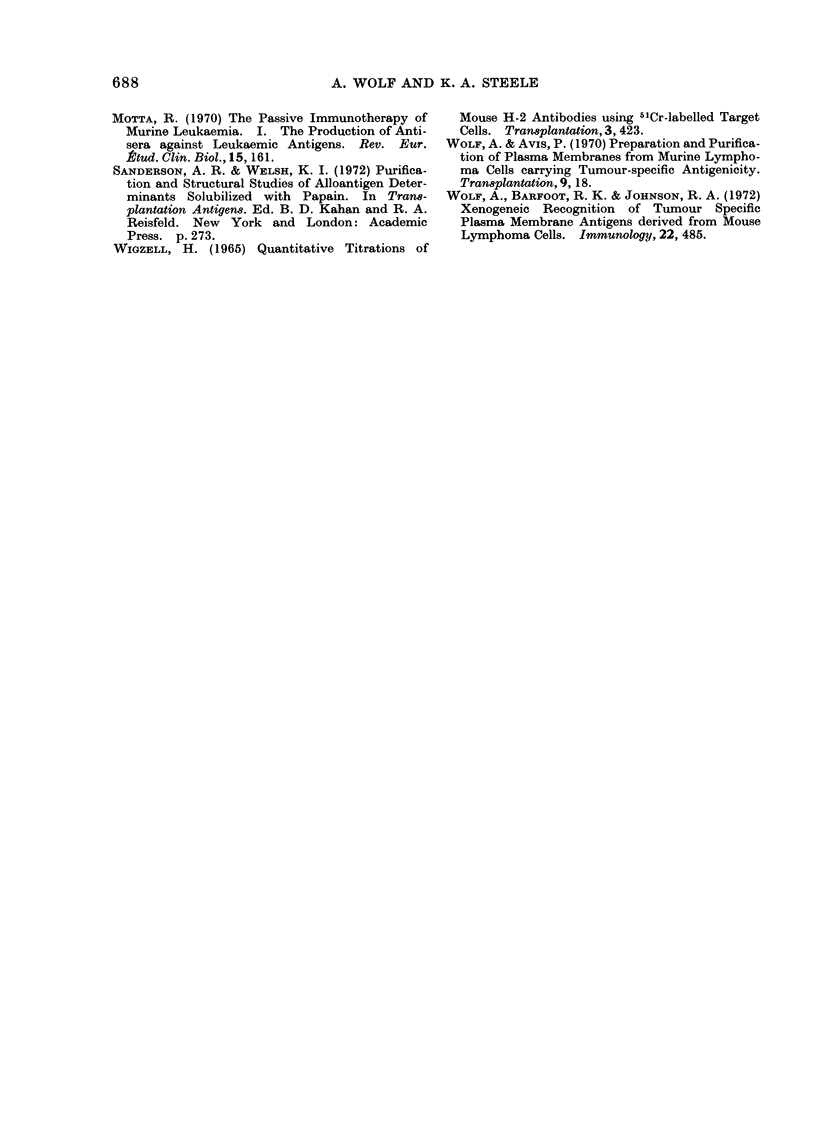# Separation of a tumour specific transplantation-type antigen from the ascitic fluid of mice bearing a syngeneic lymphoma.

**DOI:** 10.1038/bjc.1975.116

**Published:** 1975-06

**Authors:** A. Wolf, K. A. Steele


*Br. J. Cancer* (1975) **31**, 684

***Short Communication***

### SEPARATION OF A TUMOUR SPECIFIC TRANSPLANTATION-TYPE ANTIGEN FROM THE ASCITIC FLUID OF MICE BEARING A SYNGENEIC LYMPHOMA

A. WOLF AND K. A. STEELE

*From the Chester Beatty Research Institute, Institute of Cancer Research, Clifton Avenue, Belmont, Sutton, Surrey, England*

Received 27 January 1975. Accepted 7 March 1975

In a previous publication, one of us (A.W.) has described the isolation of a tumour specific transplantation antigen (TSTA) in the form of a cell membrane fraction as the basis of immunogenicity (Wolf and Avis, 1970). In this communication we report the isolation of a soluble TSTA from the ascitic fluid of mice bearing the immunogenic lymphoma SL2, the tumour specific antigenic activity being identified serologically. The procedures used for isolating the TSTA were similar to those employed for the isolation of normal transplantation antigens by other workers (cf Sanderson and Welsh, 1972). Antigenic activity was assessed by the capacity of the separated fractions to inhibit the cytotoxicity of an antiserum directed against the TSTA of the tumour cells.

### MATERIAL AND METHODS

*The tumour.*—The SL2 is a spontaneous lymphoma which arose in a male DBA/2 mouse of our own colony (Wolf, Barfoot and Johnson, 1972). The tumour grows from less than 10 cells and invariably kills 100% of the inoculated animals. Some specific antigens are likely to reside on the cell membranes since the injection of $2 \times 10^7$ irradiated SL2 cells readily protect DBA/2 mice against a subsequent challenge by $10^3$ live cells and 60% of animals immunized in this way survive a live inoculum of $10^4$ cells. Moreover, specific antibodies are produced in rabbits in response to membrane fractions of SL2 cells.

*Allogeneic immune serum.*—This antiserum was raised by the method of Motta (1970). C57Bl mice received twice $2 \times 10^7$ live SL2 cells which had been incubated and were injected together with an antiserum obtained from other C57Bl mice containing alloantibodies directed against normal DBA/2 lymphoid cell antigens.

*Monitoring and quantitation of specific antigenic activity.*—A complement dependent cytotoxic technique based on the release of $^{51}Cr$ from labelled target cells by a specific antiserum was used (Wigzell, 1965). Inhibitory tests for measuring TSTA activity were performed by preincubation of antiserum with a dilution series of fractions of ascitic fluid before adding labelled cells and preserved guinea-pig complement (Wellcome Reagents Ltd). Complement was used unabsorbed, giving at the final concentration of approximately 1 : 16 a background of 10% of the maximal $^{51}Cr$ release. Fresh weanling rabbit serum tested as complement source was less reliable.

### EXPERIMENTS AND RESULTS

### *Typing of the antiserum*

The anti-SL2 serum was titrated unabsorbed, and absorbed with normal DBA/2 lymphoid cells (v/v for 1 h) against 3 types of labelled cells (1) SL2 cells as the specific target, (2) normal DBA/2 lymphoid cells as carrier of normal DBA/2 transplantation antigens and (3) TXL9

Requests for reprints should be sent to A. Wolf at the above address.

cells (from an x-ray induced C57Bl lymphoma) as cells genetically different from DBA/2 derived cells. Figure 1 shows that after absorption the antiserum

FIG. 1.—Allogeneic anti-SL2 serum unabsorbed and absorbed with normal DBA/2 lymphoid cells titrated against $^{51}$Cr-labelled SL2 cells and normal DBA/2 lymphoid cells. The figures are means of duplicates. ○—○ unabsorbed anti-serum against SL2 cells, ▲- - -▲ absorbed anti-serum against SL2 cells, ●—● unabsorbed anti-serum against normal DBA/2 cells, ▼—▼ absorbed anti-serum against normal DBA/2 cells. 100% = total releasable label by freezing and thawing (approximately 8 × 10$^{3}$ ct/min for SL2 cells and 5 × 10$^{3}$ ct/min for normal DBA/2 cells). A background of 10% is substracted.

still exhibited considerable lytic capacity for the specific SL2 cells where the titre against normal DBA/2 cells was almost completely abolished, indicating the presense in the serum of an antibody population directed against the specific SL2-antigen. No lysis at all was observed of TXL9 cells. The absorbed antiserum was therefore considered to be a specific agent suitable for the detection of the TSTA of the SL2 lymphoma.

*Isolation of TSTA*

Figure 2 illustrates the purification procedures adopted to isolate the specific SL2-antigen. Inhibitory tests for monitoring TSTA activity were performed with all preparations, details of control tests being given under " Quantitation of TSTA ". Ascitic fluid harvested from 12–15 tumour bearing animals was centrifuged at 100,000 *g* for 2 h and at 250,000 *g* for 1 h to remove all particulate material. Of the three ammonium sulphate precipitates, only the 40–60% fraction contained considerable specific inhibitory activity. After dialysis and concentration this fraction was applied to a Biogel A 0·5 m column (32 × 500 mm). The most active fractions (2 and 3) were pooled and rechromatographed on the same type of Biogel column as before. Inhibitory fractions (consisting of material between 50,000 and 100,000 mol. wt) were pooled, dialysed against 0·005 mol Tris-phosphate buffer pH 7·8, and applied to a DEAE-cellulose column (250 × 22 mm). Elution was carried out by a linear gradient from 0·005 to 0·5 mol Tris-phosphate buffer, pH 7·8. Specific inhibitory activity was confined to fractions eluted by 0·1–0·4 mol buffer, the highest specific activity residing in fractions eluted between 0·1 and 0·2 mol buffer.

*Quantitation of TSTA*

In order to quantitate the TSTA activity, inhibitory tests were performed with five 5-fold dilutions of all preparations. The diluted fractions were mixed and incubated with absorbed antiserum diluted 1 : 100. After incubation for 1 h $^{51}$Cr-labelled specific target cells and complement were added (all components at 30 $\mu$l volumes) and incubation was continued for 45 min at 37°C. With 2 ml of Medium 199 pipetted into each tube, the cells were spun down and aliquots of supernatant were removed for counting the amount of the released $^{51}$Cr.

Two types of tests were performed to discriminate between inhibitory effects by TSTA on the one hand and by anti-

FIG. 2.—Isolation of specific inhibitory TSTA activity from the ascitic fluid of DBA/2 mice bearing the SL2 lymphoma. b.m. = buffer molarity.

complementary activity or normal transplantation antigens on the other. Complement inhibiting activity was examined by removing the mixture of ascitic fluid fraction and antiserum from the incubated test samples after sedimenting the antibody coated cells, before the addition of complement, thus avoiding contact between the latter and the fractions. If such experiments showed a higher degree of cytotoxicity than those where the same fraction remained in the test tube for the final cytotoxic reaction with complement, this was taken as evidence that the fraction interfered with the complement action. (Cytotoxicity was of the same magnitude when control anti-serum was removed after 10 min incubation with cells as when the antiserum remained in the test tube.) Almost all fractions containing material of molecular weight of more than 200,000 exhibited effects on complement and were subsequently excluded from further purification.

Tests for inhibition by normal transplantation antigens were carried out using unabsorbed antiserum and normal DBA/2 lymphoid cells. Such experiments did exhibit much less inhibition than those performed with absorbed antiserum and SL2 cells and also showed a different distribution of the inhibitory activity over the examined fractions, indicating that inhibition in the TSTA system was indeed specific.

For calculating the relative specific activity of the various fractions, 50% inhibition of the cytotoxic capacity of the control anti-serum was used as an endpoint and the protein contents (Lowry *et al.*, 1951) per ml of the preparation at this point were compared.

The purification efficiency defined in this way is demonstrated in the Table, which gives details of one of our best preparations. Although there was a loss in total activity by the purification procedure, there was a gain in specific activity

TABLE.—*Relative TSTA Activity at Different Stages of Purification of Ascitic Fluid of Mice Bearing the SL2 Lymphoma (Starting Material was the Ascitic Fluid from 12 Mice and from Approximately* $10^{10}$ *Cells)*

| Separation procedure | Total protein (mg) | Relative activity(4) |
|---|---|---|
| Crude ascitic fluid | 500 | ND(1) |
| Ammonium sulphate 40–60% | 350 | 50 |
| Biogel I Fractions 2 and 3 | 70 | 20 |
| Biogel II Fractions 2 and 3 | 13·5 | 4 |
| DEAE-cellulose | | |
| Fraction 1 (0·005–0·1)(3) | 0·1 | NI(2) |
| Fraction 2 (0·1–0·2)(3) | 0·2 | 0·2 |
| Fraction 3 (0·2–0·3)(3) | 1·5 | 1·0 |
| Fraction 4 (0·3–0·4)(3) | 3·0 | 4·0 |
| Fraction 5 (0·4–0·5)(3) | 1·0 | NI(2) |

(1) Not done (see Text).

(2) No inhibition.

(3) Molarity of eluting buffer.

(4) The amount in $\mu$g protein which reduced by 50% the cytotoxicity of 1 ml anti-serum diluted 1 : 100.

of 1 : 250 between the ammonium sulphate precipitate and the DEAE fraction 2. There was also specific activity in DEAE fraction 3. The crude ascitic fluid was highly anti-complementary and its TSTA activity could not be measured as a distinct activity. No attempts were made to absorb the anti-complementary activity from the crude starting material.

### DISCUSSION

From the results it may be concluded that a soluble TSTA-type activity is present in and can be isolated from the ascitic fluid of DBA/2 mice bearing the SL2 lymphoma.

The specific antigenicity of crude ascitic fluid could not be measured as a distinct activity because of its high content of anti-complementary material, but TSTA was already detectable in the 40–60% ammonium sulphate precipitate. Purification by Biogel and DEAE-cellulose led to a 250-fold concentration of the specific activity. When this fraction was iodinated and subjected to electrophoresis on polyacrylamide, it appeared as a single although widely distributed component in the $\beta$ region.

The fractionation shown was one of two procedures resulting in preparations of similarly good specific activity. From eight other batches of ascitic fluid preparations were obtained which contained a range of 15–50 times increased specific activity, the differences due possibly to a varying percentage of nonspecific protein in the starting material. Attempts to remove the nonspecific moiety from preparations by absorption on a cross-linked rabbit anti-mouse serum usually resulted in the doubling of the specific activity but there probably still remained a large amount of contaminating, perhaps non-antigenic or weakly antigenic, material in the fractions. More experiments to study this partly purified TSTA fraction, although difficult by the tendency of the material to aggregate, are in progress.

The authors wish to thank Professor P. Alexander for encouraging this study and Dr P. Karran for carrying out the polyacrylamide electrophoresis. This work has been supported by Grants made to the Chester Beatty Research Institute by the Cancer Research Campaign and the Medical Research Council.

### REFERENCES

LOWRY, O. H., ROSEBROUGH, N. J., FARR, L. & RANDALL, R. J. (1951) Protein Measurement with the Folin Phenol Reagent. *J. biol. Chem.*, **193**, 265.

Motta, R. (1970) The Passive Immunotherapy of Murine Leukaemia. I. The Production of Antisera against Leukaemic Antigens. *Rev. Eur. Étud. Clin. Biol.*, **15**, 161.

Sanderson, A. R. & Welsh, K. I. (1972) Purification and Structural Studies of Alloantigen Determinants Solubilized with Papain. In *Transplantation Antigens*. Ed. B. D. Kahan and R. A. Reisfeld. New York and London: Academic Press. p. 273.

Wigzell, H. (1965) Quantitative Titrations of Mouse H-2 Antibodies using $^{51}$Cr-labelled Target Cells. *Transplantation*, **3**, 423.

Wolf, A. & Avis, P. (1970) Preparation and Purification of Plasma Membranes from Murine Lymphoma Cells carrying Tumour-specific Antigenicity. *Transplantation*, **9**, 18.

Wolf, A., Barfoot, R. K. & Johnson, R. A. (1972) Xenogeneic Recognition of Tumour Specific Plasma Membrane Antigens derived from Mouse Lymphoma Cells. *Immunology*, **22**, 485.